# Comprehensive Management of a Giant Left Frontal AVM Coexisting with a Bilobed PComA Aneurysm: A Case Report Highlighting Multidisciplinary Strategies and Advanced Neurosurgical Techniques

**DOI:** 10.3390/jcm14041232

**Published:** 2025-02-13

**Authors:** Corneliu Toader, Matei Serban, Razvan-Adrian Covache-Busuioc, Mugurel Petrinel Radoi, Alexandru Vlad Ciurea, Nicolaie Dobrin

**Affiliations:** 1 Department of Neurosurgery “Carol Davila”, University of Medicine and Pharmacy, 050474 Bucharest, Romania; corneliu.toader@umfcd.ro (C.T.); petrinel.radoi@umfcd.ro (M.P.R.); prof.avciurea@gmail.com (A.V.C.); 2 Department of Vascular Neurosurgery, National Institute of Neurology and Neurovascular Diseases, 077160 Bucharest, Romania; 3 Puls Med Association, 051885 Bucharest, Romania; 4 Neurosurgery Department, Sanador Clinical Hospital, 010991 Bucharest, Romania; 5 Medical Section, Romanian Academy, 010071 Bucharest, Romania; 6 “Nicolae Oblu” Clinical Hospital, 700309 Iasi, Romania; dobrin_nicolaie@yahoo.com

**Keywords:** arteriovenous malformation, posterior communicating artery aneurysm, cerebrovascular anomalies, advanced imaging, microsurgical resection, multidisciplinary approach, high-flow dynamics, hemodynamic stability

## Abstract

**Background:** Arteriovenous malformations (AVMs) are high-risk cerebrovascular anomalies that can lead to devastating complications, especially when associated with intracranial aneurysms. Their coexistence poses unique challenges in diagnosis and management due to heightened hemodynamic stress and rupture risks. This case presents a 35-year-old woman with a giant unruptured left frontal AVM and a bilobed posterior communicating artery (PComA) aneurysm, highlighting the critical role of advanced imaging, meticulous surgical planning, and individualized care in addressing complex cerebrovascular conditions. **Methods:** The patient presented with a generalized tonic–clonic seizure, her first-ever neurological event. Advanced imaging, including digital subtraction angiography and 3D rotational imaging, revealed a 3–4 cm AVM supplied by the left middle and anterior cerebral arteries, with venous drainage into the superior sagittal sinus. Additionally, an unruptured bilobed PComA aneurysm was identified. Given the AVM’s large size, high-flow dynamics, and significant rupture risk, surgical resection was prioritized. The aneurysm, being stable and anatomically distinct, was managed conservatively. Microsurgical techniques were employed to ensure complete AVM resection while preserving critical vascular and neurological structures. **Results:** Postoperative angiography confirmed the complete removal of the AVM without residual nidus or abnormal vascular connections. The patient recovered without complications, achieving seizure freedom and preserved neurological function. At the three-month follow-up, imaging showed a stable resection cavity and a hemodynamically stable aneurysm. **Conclusions:** This case demonstrates the power of multidisciplinary care and advanced neurosurgical techniques in achieving curative outcomes for complex cerebrovascular anomalies. It underscores the importance of risk-prioritized strategies and highlights emerging directions for the field, including AI-integrated imaging, hybrid treatment approaches, and long-term studies on hemodynamic stability post-resection. This case contributes valuable insights into optimizing outcomes for patients with coexisting AVMs and aneurysms, offering hope for those facing similarly challenging diagnoses.

## 1. Introduction

Arteriovenous malformations (AVMs) represent rare cerebrovascular anomalies defined by direct arterial-to-venous shunting, bypassing normal capillary networks. Detected at a rate of 1 per 100,000 individuals annually, AVMs carry significant clinical implications due to their unpredictable nature [1]. Though often asymptomatic, larger or high-flow AVMs pose substantial risks, including rupture, intracerebral hemorrhage, and progressive neurological deterioration. Hemorrhage remains the most feared complication, with an annual risk of 2% to 4%, carrying mortality rates as high as 29% following rupture. Up to 55% of survivors face long-term disability, highlighting the critical need for early detection and intervention [2,3].

AVMs are more common in males, while intracranial aneurysms—a related vascular anomaly—show a higher prevalence in females, with a 3:2 female-to-male ratio. Intracranial aneurysms occur in 1% to 5% of the general population, with rupture leading to subarachnoid hemorrhage in a significant proportion of cases [4,5]. When AVMs and aneurysms coexist, their combined hemodynamic stress exacerbates the rupture risk, presenting unique diagnostic and therapeutic challenges. Managing such cases demands a tailored multidisciplinary approach to balance the surgical risks and long-term patient outcomes [6].

Recent advancements in imaging technologies have transformed the evaluation of cerebrovascular anomalies. High-resolution MRI, digital subtraction angiography (DSA), and 3D rotational angiography now allow unparalleled visualization of vascular structures, flow dynamics, and anatomical relationships [7]. These tools, combined with evolving surgical and endovascular techniques, have significantly improved diagnostic precision and treatment outcomes. New embolization strategies and hybrid procedures have expanded options for complex cases, particularly those involving concurrent vascular pathologies [8].

This report describes a 35-year-old woman with a giant unruptured left frontal AVM coexisting with a bilobed posterior communicating artery (PComA) aneurysm, discovered incidentally after a generalized tonic–clonic seizure. The coexistence of AVMs and aneurysms presents substantial diagnostic and therapeutic challenges due to their combined hemodynamic stress and elevated risk of rupture. The sudden onset of seizures highlights the critical need for early detection and comprehensive evaluation of both structural and functional abnormalities. In this case, thorough imaging, clinical assessment, and individualized care planning allowed for an optimal treatment strategy that prioritized the patient’s safety.

Advanced imaging using DSA and 3D rotational angiography enabled precise mapping of the AVM’s vascular architecture and the aneurysm’s characteristics, guiding the decision to perform surgical resection of the AVM while conservatively monitoring the aneurysm. This case emphasizes how multidisciplinary collaboration between neurosurgeons, radiologists, and neurologists is essential in managing complex vascular anomalies. The patient’s successful outcome—seizure freedom, neurological stability, and no postoperative complications—demonstrates the effectiveness of combining meticulous preoperative planning with personalized treatment approaches. By presenting this case, we aim to provide practical insights into the decision-making processes, risk assessment, and technical considerations that are essential when managing patients with multiple vascular pathologies.

## 2. Case Report

A 35-year-old woman presented to our clinic after experiencing her first generalized tonic–clonic seizure. The event, which occurred two weeks prior, was sudden and unexpected, interrupting a history of apparent good health. According to her account, the seizure lasted approximately two minutes and was followed by a prolonged postictal phase characterized by confusion and fatigue, persisting for several hours. She denied any prodromal symptoms, such as headaches, visual disturbances, or focal neurological deficits in the days or weeks preceding the event.

Her medical history was unremarkable, with no previous diagnosis of seizures, neurological disorders, or systemic illnesses. Likewise, there was no familial predisposition to epilepsy or cerebrovascular abnormalities. She reported no use of alcohol, tobacco, or recreational drugs, and her lifestyle appeared conducive to maintaining good health. This abrupt neurological event in an otherwise asymptomatic individual raised the possibility of an underlying structural or vascular abnormality and warranted further investigation. The coexistence of two distinct vascular abnormalities—a large high-flow AVM and a separate aneurysm—poses a unique clinical challenge that underscores the importance of comprehensive diagnostic imaging. This rare combination not only highlights the complexity of the patient’s condition but also provides an opportunity to explore tailored management strategies in such intricate cases.

### 2.1. Neurological Examination on Admission

On initial assessment, the patient was alert, oriented, and fully cooperative. Cognitive faculties were intact, with no signs of memory impairment, language dysfunction, or attentional deficits. A detailed neurological examination yielded the following findings:

Cranial nerves: All cranial nerve functions were normal. The pupils were bilaterally equal and reactive to light. The extraocular movements were smooth and full, with no nystagmus observed. The visual fields were intact to confrontation testing, and there were no deficits in facial symmetry, sensory modalities, or tongue movement.

Motor system: The muscle strength was robust, graded 5/5 symmetrically across all major muscle groups. The muscle tone and bulk were normal, with no evidence of spasticity, atrophy, or fasciculations. The deep tendon reflexes were brisk but symmetric, with no pathological reflexes such as clonus or a positive Babinski sign.

Sensory system: A fine and crude touch, pinprick, vibration, and proprioception were preserved throughout the upper and lower extremities. No sensory level or focal deficits were identified.

Coordination and gait: The patient performed coordination tasks, including finger-nose and heel–shin maneuvers, with precision and speed. Her gait was steady and fluid, and tandem walking revealed no ataxia or instability.

Postictal residuals: Remarkably, there were no detectable residual neurological deficits, and her neurological status had fully returned to baseline.

Although her examination was unremarkable, the sudden onset of a seizure in a previously healthy individual prompted an extensive diagnostic evaluation to investigate potential structural or vascular causes.

### 2.2. Diagnostic Workup

Given the absence of focal neurological deficits on examination and the sudden onset of a seizure in an otherwise healthy individual, advanced imaging was deemed essential to uncover the potential underlying structural or vascular abnormalities. The patient’s diagnostic evaluation began with a native and contrast-enhanced cranial CT scan, which provided the first crucial insight into the underlying pathology. This imaging revealed a serpiginous malformation within the left frontal lobe that exhibited intense contrast enhancement, findings strongly suggestive of a cerebral AVM. Despite the prominent vascular features of the lesion, the surrounding brain parenchyma appeared entirely normal, with no evidence of ischemia, hemorrhage, or edema. Additionally, the cerebroventricular system was intact and symmetrical, showing no signs of hydrocephalus or mass effect. These findings highlighted the need for more detailed vascular imaging to delineate the lesion’s anatomical characteristics and hemodynamic profile, setting the stage for targeted management planning.

**Figure 1:**
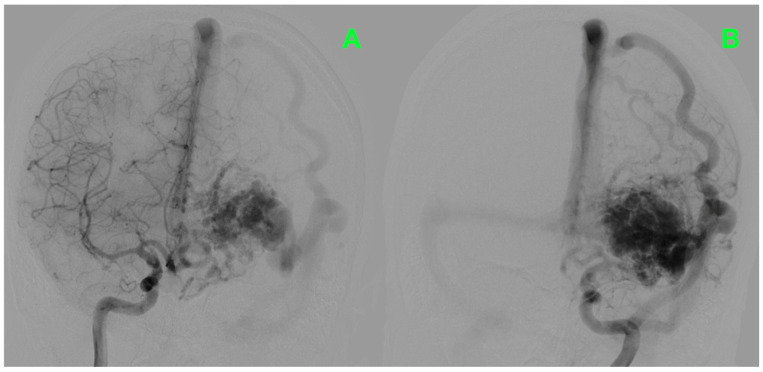
Preoperative bilateral carotid angiography. (**A**) Frontal view showing arterial feeders from the left middle and anterior cerebral arteries. (**B**) Lateral view highlighting venous drainage into the superior sagittal and transverse sinuses.

To refine the diagnosis, bilateral carotid and vertebral angiography was performed, which vividly delineated the AVM’s complex vascular anatomy and dynamic flow characteristics (Figure 1). The study confirmed the presence of a high-flow high-pressure AVM occupying the left frontal lobe, measuring approximately 3–4 cm in diameter. The arterial supply to the lesion arose from prominent feeders originating from the left middle and anterior cerebral arteries, clearly visualized in the frontal projection (Figure 1A). The venous outflow, as shown in the lateral projection, drained into the superior sagittal and left transverse sinuses through markedly dilated veins (Figure 1B). These features revealed not only the hemodynamic complexity of the lesion but also the significant risks associated with its size and high-flow nature, warranting further exploration of its structural relationships.

**Figure 2:**
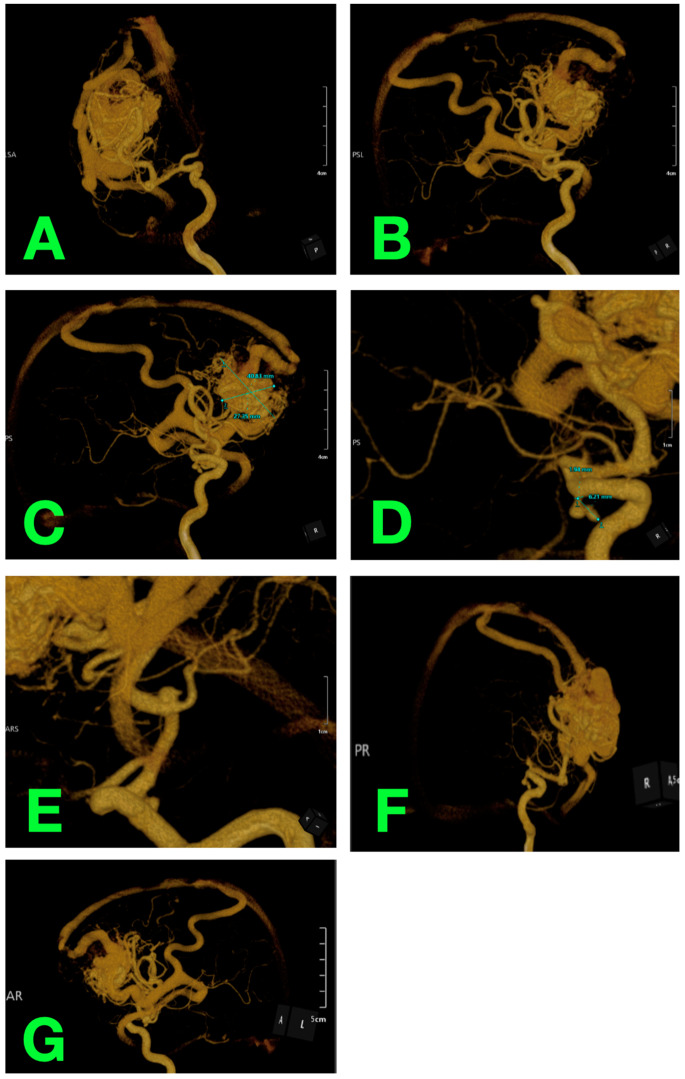
Three-dimensional rotational angiography. (**A**–**C**) Volumetric view of the AVM and its dimensions. (**D**,**E**) Close-up of the bilobed PComA aneurysm. (**F**,**G**) Dynamic flow characteristics of the AVM, emphasizing its high-pressure nature.

For a more comprehensive understanding of the AVM’s architecture and spatial relationships, 3D rotational angiography was employed (Figure 2). This advanced imaging modality provided a strikingly detailed visualization of the lesion. The AVM’s nidus, measured at 40.81 mm × 27.45 mm, was intricately interconnected with arterial feeders and draining veins (Figure 2A–C). Additionally, the 3D angiographic reconstructions identified a separate vascular abnormality—a bilobed unruptured aneurysm arising from the left PComA (Figure 2D,E). Measuring 6.21 mm in diameter, the aneurysm was anatomically distinct and hemodynamically unrelated to the AVM. The dynamic flow patterns captured in Figure 2F,G illustrated the considerable pressure within the AVM, highlighting the urgent need for intervention to mitigate its associated risks.

**Figure 3:**
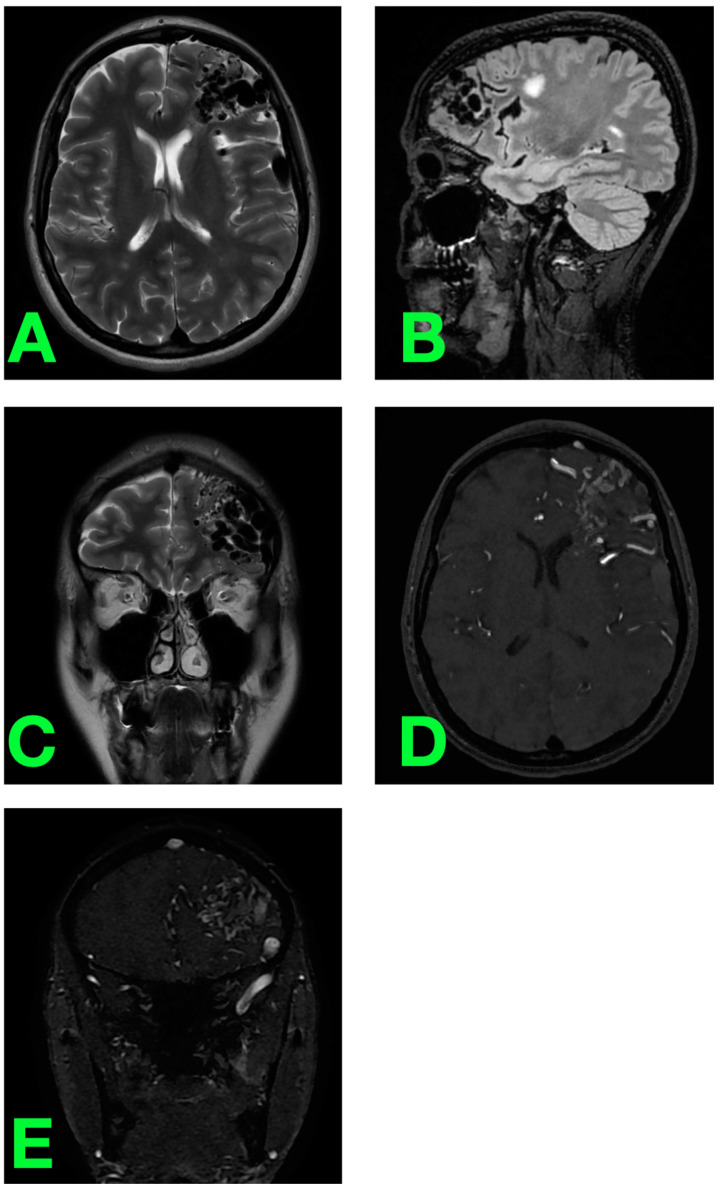
Preoperative MRI. (**A**–**C**) T2-weighted axial, sagittal, and coronal images showing the AVM nidus and its flow void pattern. (**D**,**E**) Post-contrast sequences illustrating enhancement of the nidus and feeding arteries.

Complementing these findings, the MRI of the brain provided further detail regarding the AVM’s structural and spatial context (Figure 3). T2-weighted axial imaging demonstrated a prominent flow void pattern within the left frontal lobe, corresponding to the AVM nidus (Figure 3A). Sagittal T2-weighted imaging revealed the lesion’s relationship to the adjacent brain structures, showing no evidence of mass effect or perilesional edema (Figure 3B). Coronal T2-weighted sequences emphasized the complex vascular morphology of the AVM, allowing for an appreciation of its intricate architecture (Figure 3C). Post-contrast imaging, particularly the axial and coronal views, displayed robust enhancement of the AVM’s nidus and arterial feeders, with no evidence of inflammatory or ischemic changes in the surrounding tissue (Figure 3D,E). These MRI findings complemented the angiographic results, offering a nuanced view of the lesion’s anatomy and further confirming its high-risk profile.

Notably, while the angiographic studies and 3D imaging confirmed the presence of a bilobed unruptured aneurysm of the left posterior communicating artery, it was deemed stable and unlikely to pose an immediate threat. This allowed the focus to remain on addressing the AVM, which presented a more significant risk to the patient’s neurological function and overall prognosis. No additional vascular abnormalities were identified, and the structural integrity of the remaining cerebral vasculature appeared preserved.

### 2.3. Clinical Impression and Decision Making

The coexistence of a giant unruptured AVM and a bilobed PComA aneurysm presented a unique and challenging clinical scenario. The AVM, characterized by its large size, left frontal location, and high-flow dynamics, posed a substantial risk of rupture or progressive neurological deterioration. After careful deliberation, a multidisciplinary team determined that surgical resection of the AVM was the most prudent course of action. Given the patient’s young age and the AVM’s high-risk features, early intervention offered the best chance of preventing a catastrophic outcome.

The combination of detailed imaging and clinical evaluation revealed a unique and high-risk scenario. The AVM’s size, high-flow nature, and cortical location necessitated a tailored surgical approach, as it posed an imminent risk of rupture or neurological compromise. Simultaneously, the stable yet anatomically distinct aneurysm presented a separate vascular pathology requiring surveillance. The absence of hemorrhage or other acute complications allowed for deliberate planning to address the AVM as the primary threat.

The patient’s unremarkable neurological examination juxtaposed with the severity of the radiological findings underscored the importance of comprehensive imaging in asymptomatic or minimally symptomatic presentations. This case exemplifies the need for multidisciplinary collaboration and individualized treatment planning in complex cerebrovascular disorders.

The aneurysm, being anatomically and hemodynamically distinct from the AVM, was deemed suitable for conservative monitoring. To assess the stability and rupture risk of the bilobed PComA aneurysm, we applied the PHASES score, an established risk assessment tool for intracranial aneurysms. The PHASES score integrates factors such as patient age, aneurysm size, location, hypertension, and history of subarachnoid hemorrhage to estimate the likelihood of rupture. In this case, the patient’s PHASES score was calculated as 1, reflecting an annual rupture risk of approximately 0.2%. The aneurysm’s size (<7 mm), the absence of irregular wall morphology, and the low-risk PHASES score supported the decision for conservative management. By prioritizing the AVM’s surgical treatment while deferring aneurysm intervention, the approach minimized unnecessary surgical risk while addressing the most immediate threat. This tailored decision exemplifies the importance of individualized strategies in the management of complex vascular anomalies.

### 2.4. Surgical Intervention

The surgical resection of the giant left frontal AVM was meticulously planned and executed under general anesthesia with orotracheal intubation. Recognizing the hemodynamic complexity of the AVM, the surgical strategy was designed to prioritize complete nidus excision, while preserving neurological function and minimizing intraoperative risks. A multidisciplinary team, including senior neurosurgeons, anesthesiologists, and neurophysiologists, ensured a seamless integration of expertise during the procedure.

The procedure commenced with a left parasagittal frontoparietal craniotomy, meticulously crafted to optimize access while minimizing cortical disruption. The bone flap was carefully elevated, and the dura mater was incised semicircularly, preserving a pedicle attachment to the superior sagittal sinus to maintain venous integrity.

**Figure 4:**
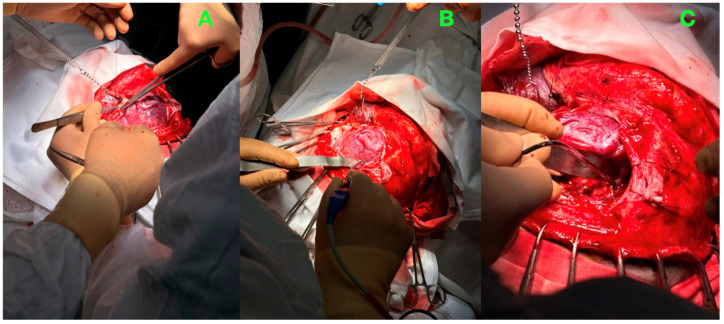
Intraoperative observations of AVM resection. (**A**): The initial exposure of the left frontal AVM following a carefully crafted parasagittal frontoparietal craniotomy. The dura mater has been reflected, revealing the prominent cortical draining vein and the AVM’s vascular network. This image demonstrates the precision of the surgical exposure, highlighting the surrounding cortical tissue, which was preserved during the procedure. (**B**): The stepwise dissection of the AVM’s arterial feeders using microdissection techniques and high-magnification visualization. The feeding arteries are systematically coagulated and divided to progressively devascularize the nidus. The surrounding cortical vessels remain intact, underscoring the meticulous preservation of the normal brain vasculature. (**C**): The resection cavity after complete en bloc excision of the AVM. The cortical draining vein has visibly collapsed, indicating the cessation of abnormal arteriovenous shunting. The surgical field is hemostatic, with no evidence of residual nidus or bleeding, reflecting the precision of the procedure and thorough intraoperative inspection.

The intraoperative images (Figure 4) highlight critical stages of the intervention, capturing the complexity of the AVM’s high-flow dynamics and the precision required to navigate its proximity to eloquent cortical regions. These photographs serve to illustrate the methodical progression of the resection, from initial exposure and vascular dissection to the successful removal of the nidus.

Upon dural reflection, the AVM’s cortical draining vein was immediately visualized as a tense reddish vessel—a hallmark of high-flow shunting. Guided by magnified visualization and neuronavigation, dissection proceeded along the medial surface of the precentral gyrus, exposing the nidus. The arterial feeders were systematically identified, coagulated, and divided, employing a stepwise, circumferential approach to avoid sudden hemodynamic shifts. High-magnification visualization allowed for precise dissection, ensuring that the normal vasculature remained untouched.

The cortical draining vein, although prominent, was strategically preserved during the initial phases of resection to maintain the outflow until the nidus was fully devascularized. This approach prevented venous congestion and minimized the risk of intraoperative hemorrhage. Once all feeders were securely occluded, and the nidus was completely isolated, the draining vein visibly collapsed, marking the cessation of abnormal shunting.

Hemostasis was meticulously achieved using bipolar cautery and advanced hemostatic agents, including Surgicel^®^ and flowable hemostats, ensuring a dry surgical field. The resection cavity was inspected for residual vascular connections, and the absence of active bleeding or abnormal vessels was confirmed. A duraplasty was performed to reconstruct the dura mater, maintaining a watertight closure. The bone flap was repositioned and secured, and the scalp was sutured in layers to ensure optimal wound healing. An epidural drain was placed for postoperative monitoring and removed within 48 h.

The high-flow dynamics of the AVM demanded constant vigilance during feeder ligation to prevent abrupt hemodynamic changes. The proximity to eloquent motor regions necessitated a balance between the aggressive nidus resection and the preservation of functional brain tissue. Despite these challenges, the surgical plan was executed flawlessly, with no compromise to adjacent cortical or vascular structures.

### 2.5. Postoperative Course

The patient was transferred to the neurosurgical intensive care unit (ICU) immediately following the successful resection of the left frontal AVM. Extubation was achieved within minutes after surgery, as the patient exhibited rapid recovery of spontaneous ventilation, stable hemodynamics, and intact protective reflexes. Upon extubation, the patient was fully alert, cooperative, and oriented to time, place, and person, with no signs of neurological impairment.

The neurological examination conducted shortly after extubation revealed an optimal outcome. The motor strength was graded as 5/5 in all extremities, with symmetrical reflexes and preserved sensation across all dermatomes. The cranial nerve function was intact, with normal pupillary responses, smooth extraocular movements, and symmetrical facial expression. The patient demonstrated clear speech and was able to follow commands without difficulty, indicating the absence of cortical or brainstem dysfunction. Despite the complexity of the lesion and its proximity to eloquent regions, no deficits were observed in coordination, gait, or sensory processing.

### 2.6. Initial Imaging and Clinical Monitoring

**Figure 5:**
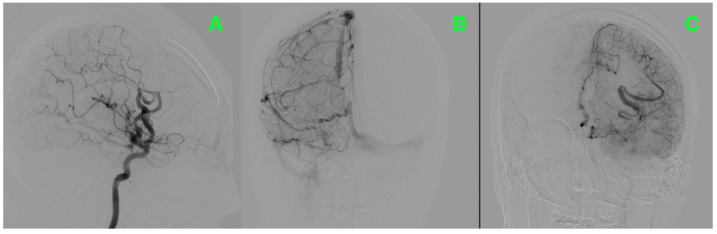
Postoperative DSA confirmed complete resection of the AVM with no evidence of residual nidus or abnormal vascular structures. (**A**): This demonstrates the absence of the AVM nidus and feeding arteries, with no abnormal arteriovenous connections observed. (**B**): This highlights the normalization of the vascular flow in the left frontal region, with the superior sagittal sinus and surrounding cortical vessels remaining intact. (**C**): This provides a detailed view of the resection site, confirming the resolution of the high-flow dynamics previously associated with the AVM.

**Figure 6:**
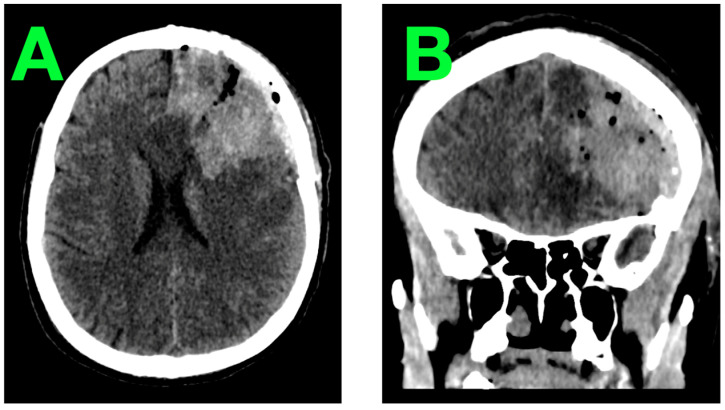
Postoperative cranial CT provided additional confirmation of a favorable outcome, showing a well-defined resection cavity without complications. (**A**): This displays the resection cavity in the left frontal lobe, with no evidence of residual nidus, hemorrhage, or edema. (**B**): This confirms the preservation of midline structures, with no mass effect, hydrocephalus, or ischemic changes. The cerebroventricular system remains symmetrical and normal.

An immediate postoperative DSA (Figure 5) and cranial CT (Figure 6) scan were performed within two hours of surgery to evaluate the resection site and ensure the absence of complications.

The hemodynamic parameters, including the blood pressure, heart rate, and oxygen saturation, were stable throughout the postoperative period. No signs of elevated intracranial pressure or systemic complications were detected during monitoring.

The patient reported no significant pain or discomfort following the procedure, reflecting the precision of the surgical intervention. The surgical wound was clean and dry, with no evidence of cerebrospinal fluid leakage or infection. The epidural drain yielded only minimal serosanguinous fluid, and no active bleeding was observed.

Antiepileptic therapy (Levetiracetam 500 mg twice daily) was initiated, and the patient tolerated it well. Given her stable neurological and systemic condition, early mobilization was planned for postoperative day one to facilitate recovery.

The patient returned for a scheduled follow-up three months after the surgical resection of the giant left frontal AVM. During this period, the patient experienced significant neurological improvement, with continued seizure freedom, enhanced cognitive function, and increased physical stamina, leading to improved performance in daily activities and the absence of new symptoms.

**Figure 7:**
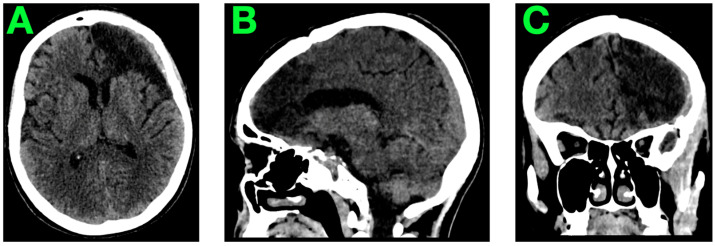
Three-month follow-up CT. (**A**): This view demonstrates a hypodense area in the left frontal lobe, corresponding to the resection cavity from the excision of the AVM. The cavity appears well-defined with no evidence of residual nidus, hemorrhage, or ischemia. The surrounding parenchyma is preserved, with no signs of edema or other pathological changes. (**B**): The sagittal section highlights the absence of mass effect or midline shift. The resection cavity is visible, with well-preserved anatomical relationships in the adjacent regions. The cerebral parenchyma and midline structures remain intact, reflecting a stable postoperative condition. (**C**): The coronal view confirms the integrity of the cerebroventricular system, with no evidence of hydrocephalus or other abnormalities. The surgical site is clean, and there are no signs of postoperative complications, such as venous congestion or infarction.

A follow-up native cranial CT scan was performed to evaluate the surgical site and surrounding brain structures (Figure 7). The imaging findings were consistent with an excellent postoperative outcome.

The follow-up imaging confirmed a stable resection cavity with no evidence of residual AVM or aneurysm progression. The patient remained seizure-free and continued to demonstrate a stable neurological status, reflecting the precision of the surgical intervention and effective perioperative management.

The surgical intervention successfully addressed the AVM without complications, achieving complete resection and preserving the patient’s neurological function. The imaging and clinical evaluations demonstrated excellent long-term stability, underscoring the precision of the surgical approach and the effectiveness of the postoperative care. This case highlights the importance of a multidisciplinary strategy in managing complex AVMs, resulting in a favorable clinical and functional outcome.

## 3. Discussion

This case highlights the successful management of a giant unruptured frontal AVM coexisting with a bilobed PComA aneurysm, underscoring the complexity and rarity of such presentations. While AVMs and intracranial aneurysms are individually well-documented, their coexistence creates unique hemodynamic and clinical challenges that require meticulous planning and multidisciplinary collaboration. The patient’s minimal symptoms, presenting only as a generalized tonic–clonic seizure, contrasted with the severity of the underlying vascular abnormalities, emphasizing the critical role of advanced imaging and individualized treatment strategies in cerebrovascular care.

### 3.1. Epidemiology and Pathophysiological Context

AVMs are rare, with an annual detection rate of 1 per 100,000 individuals and a prevalence of 18 per 100,000. They are more common in males and typically present in the second to fourth decades of life [9]. Hemorrhagic stroke remains the most severe complication, with a 2–4% annual rupture risk. The mortality following rupture reaches up to 30%, and long-term disability impacts a significant proportion of survivors [10]. Aneurysms are present in 2–17% of AVM cases, arising from arterial wall stress caused by high-flow shunting. This pathophysiological interplay emphasizes the need to address the hemodynamic burden imposed by AVMs to mitigate the secondary vascular risks [11].

In this case, the aneurysm was anatomically distinct and hemodynamically stable, allowing prioritization of the AVM. The decision reflects evidence that addressing the primary pathology often normalizes the arterial flow, reducing the rupture risk of the associated aneurysms. Comprehensive imaging revealed the extent of the vascular anomalies, guiding a strategy that minimized the risk while achieving curative outcomes.

### 3.2. Advances in Imaging and Their Role in Management

Imaging played a pivotal role in this case, providing detailed insights into the AVM’s architecture, flow dynamics, and spatial relationships. The DSA enabled precise identification of the feeding arteries and draining veins, while the 3D rotational imaging enhanced the spatial resolution for surgical planning [12]. Functional MRI and perfusion studies, although not employed here, could offer additional benefits in assessing AVMs near eloquent cortical regions or estimating hemodynamic stress [13,14].

Intraoperatively, tools like indocyanine green angiography provide real-time confirmation of nidus obliteration and flow normalization [15]. These technologies, combined with neuronavigation and neurophysiological monitoring, optimize surgical precision and minimize complications, particularly in high-risk lesions near eloquent regions [16].

### 3.3. Treatment Modalities and Decision Making

The management of AVMs requires a tailored approach, integrating factors such as size, Spetzler–Martin grade, hemodynamic risk, and patient-specific considerations. Options include microsurgical resection, endovascular embolization, stereotactic radiosurgery, or combinations of these modalities [3].

Microsurgical resection remains the definitive treatment for large high-flow AVMs, particularly those located in surgically accessible areas. This approach offers immediate nidus removal, eliminating the risk of rupture and normalizing the cerebral hemodynamics [17,18]. In this case, a stepwise arterial feeder ligation, combined with cortical vein preservation until complete devascularization, ensured hemodynamic stability throughout the procedure. Postoperative angiography confirmed complete resection with no residual nidus, highlighting the success of this approach.

Endovascular embolization is a valuable adjunct for AVM management, reducing nidus flow and facilitating safer resections. Advances in embolic agents such as Onyx and PHIL allow precise feeder occlusion [19]. However, embolization alone is rarely curative for large AVMs, with incomplete obliteration and recurrence risks exceeding 30% [20]. The AVM’s size and complexity in this case made direct surgical resection a more viable option, avoiding the risks associated with embolization.

Radiosurgery is effective for smaller AVMs but less suited for large or high-flow lesions due to delayed obliteration effects, which leave patients at risk of rupture during the latency period [21,22]. Additionally, AVMs larger than 3 cm exhibit lower obliteration rates and higher complication risks, including radiation-induced necrosis [23]. The AVM in this case exceeded the size threshold for radiosurgery, reinforcing the need for immediate and definitive intervention through surgery.

Hybrid approaches combining embolization and surgery are emerging as promising strategies for large AVMs. Preoperative embolization can reduce intraoperative bleeding, while staged embolization–radiosurgery may enhance obliteration in certain cases. While not employed here, these strategies may become increasingly relevant as embolic agents and imaging technologies advance [24,25].

Aneurysms associated with AVMs require careful evaluation to determine whether they are hemodynamically linked to the AVM or independent vascular anomalies. In this case, the aneurysm’s stable morphology and lack of rupture-prone features supported a conservative monitoring strategy. Evidence suggests that AVM resection often reduces aneurysm rupture risk by normalizing the vascular flow. Long-term imaging surveillance remains critical to ensuring aneurysm stability and patient safety [26].

### 3.4. Postoperative Outcomes and Long-Term Considerations

The patient’s postoperative course was exemplary, with complete AVM resection, no neurological deficits, and seizure freedom at three months. Imaging confirmed a stable resection cavity with no evidence of residual AVM or aneurysm progression. These outcomes align with reports demonstrating favorable results for large AVM resections, emphasizing the importance of tailored surgical planning and meticulous execution.

Postoperative care focused on seizure prophylaxis, early mobilization, and regular imaging surveillance. Long-term monitoring of the aneurysm ensures continued safety, reflecting best practices in cerebrovascular management. This case reinforces the potential for excellent outcomes in complex vascular anomalies with a multidisciplinary patient-specific approach.

Table 1 summarizes the existing literature on cases of AVMs and their association with PComA aneurysms or similar vascular anomalies. Each case highlights the diversity in patient demographics, vascular presentations, clinical symptoms, and therapeutic interventions. The rarity of such presentations, combined with the complexity of their management, underscores the importance of detailed imaging, multidisciplinary approaches, and individualized treatment strategies. Comparing these cases provides a deeper understanding of potential challenges and outcomes, offering valuable insights for clinicians managing similar scenarios.

### 3.5. Ethical Considerations and Risk Stratification

Risk stratification tools, such as the Spetzler–Martin grading system, guide treatment decisions by balancing surgical risks with potential benefits [32]. This case highlights the ethical challenges of treating minimally symptomatic patients with complex vascular anomalies, where the intervention risks must be weighed against the rupture risks. The decision to prioritize the AVM while monitoring the aneurysm reflects a judicious balance of safety and efficacy.

### 3.6. Future Directions

The management of AVMs and associated aneurysms continues to evolve. Emerging hybrid surgical–endovascular techniques and novel embolic agents hold promise for improving outcomes in complex cases. Advances in AI-driven imaging and computational flow modeling could enable more accurate rupture risk prediction and personalized treatment strategies. Future research should focus on the long-term hemodynamic effects of AVM resection on associated aneurysms and exploring minimally invasive options to expand the treatment applicability.

## 4. Conclusions

This case highlights the extraordinary potential of modern cerebrovascular management, demonstrating how the integration of advanced imaging, meticulous surgical precision, and personalized treatment strategies can achieve remarkable outcomes in even the most complex scenarios. The coexistence of a giant unruptured left frontal AVM and a bilobed PComA aneurysm presented a formidable challenge, requiring innovative thinking and a multidisciplinary approach. By prioritizing the AVM’s surgical resection while conservatively monitoring the stable aneurysm, the management strategy reflected a calculated balance of risk and benefit, tailored specifically to the patient’s condition. The decision-making process emphasized the importance of addressing the most immediate threat while avoiding unnecessary interventions.

The success of this case is evident not only in the technical achievement of a complete AVM resection but also in the profound impact on the patient’s quality of life. Seizure freedom, preserved neurological function, and stable postoperative imaging underscore the transformative power of precise individualized care. Beyond the operating room, this case exemplifies how a thoughtful and collaborative approach can translate into real-world benefits for patients, providing them with improved functionality and a renewed sense of normalcy.

As the field of cerebrovascular medicine continues to evolve, this case underscores several key directions for future innovation and research. Advances in imaging technology, particularly the integration of artificial intelligence, hold the potential to revolutionize the way clinicians assess AVMs and aneurysms. AI-driven hemodynamic modeling and risk prediction could provide unprecedented accuracy, enabling clinicians to refine treatment plans and anticipate complications with greater confidence. Similarly, hybrid approaches combining embolization, radiosurgery, and minimally invasive surgical techniques represent an exciting frontier, particularly for high-risk or surgically inaccessible lesions. The development of novel embolic agents and surgical tools may further expand the range of treatable cases, improving safety and outcomes for patients.

Long-term follow-up strategies for patients with coexisting vascular anomalies also merit further study. Understanding the hemodynamic changes that occur after AVM resection, particularly their impact on associated aneurysms, is critical for refining surveillance protocols and optimizing the timing for additional interventions. Additionally, future research should prioritize patient-centered metrics, examining long-term functional recovery, quality of life, and psychological well-being. These efforts will ensure that clinical success is measured not only by technical outcomes but also by meaningful improvements in patients’ lives.

This case is more than an example of effective cerebrovascular treatment—it is a testament to the remarkable progress in neurosurgery and the promise of future innovation. It demonstrates the importance of collaboration across disciplines, from imaging specialists to neurosurgeons, in addressing complex vascular anomalies with precision and care. By pushing the boundaries of what is possible, we continue to redefine the standard of care, ensuring that even the most challenging cases are met with optimism, expertise, and the tools needed to deliver the best possible outcomes.

## Figures and Tables

**Table 1:** This table consolidates the published reports of AVMs with associated PComA aneurysms or comparable cerebrovascular conditions. The columns outline patient age, gender, vascular anomalies, clinical presentations, chosen interventions, and outcomes, emphasizing the nuanced decision making required in each case. Management strategies range from conservative approaches for stable lesions to microsurgical and endovascular interventions for high-risk anomalies.

Reference	Age	Gender	Vascular Anomalies	Presentation	Intervention	Outcome
[27]	15	Male	Pure arterial malformation (PAM) of PComA compressing optic tract	Progressive visual field impairment	Microsurgical clipping of PComA	Symptom progression ceased; gradual visual improvement
[28]	16	Female	PAM involving left PCA and PComA; associated with hippocampal dysplasia	Uncontrolled seizures	Conservative management with anti-seizure medications	Seizure-free at one-year follow-up
[29]	40	Male	PAM involving right supraclinoid ICA and right pericallosal artery; non-visualization of left ICA	Intermittent headaches; transient aphasia	Conservative management	Symptomatic improvement at four-month follow-up
[30]	60	Female	Ruptured PComA aneurysm presenting as isolated subdural hematoma	Acute headache; altered consciousness	Surgical clipping of aneurysm	Favorable recovery post-surgery
[31]	35	Male	Distal PICA aneurysm associated with cerebellar AVM	Sudden onset headache; cerebellar signs	Endovascular coiling of aneurysm; surgical resection of AVM	Successful obliteration of aneurysm and AVM; good recovery

## Data Availability

Data are available upon reasonable request from the corresponding authors.
